# The SARAH Domain of RASSF1A and Its Tumor Suppressor Function

**DOI:** 10.1155/2012/196715

**Published:** 2012-04-09

**Authors:** Claudia Dittfeld, Antje M. Richter, Katrin Steinmann, Antje Klagge-Ulonska, Reinhard H. Dammann

**Affiliations:** ^1^AWG Tumor Genetics of the Medical Faculty, Martin-Luther-University Halle-Wittenberg, 06108 Halle, Germany; ^2^OncoRay, National Center for Radiation Research in Oncology, Medical Faculty Carl Gustav Carus, University of Technology, 06108 Halle, Dresden, Germany; ^3^Institute for Genetics, Justus-Liebig University Giessen, 35392 Giessen, Germany

## Abstract

The Ras association domain family 1A (RASSF1A) tumor suppressor encodes a Sav-RASSF-Hpo domain (SARAH), which is an interaction domain characterized by hWW45 (dSAV) and MST1/2 (dHpo). In our study, the interaction between RASSF1A and RASSF1C with MST1 and MST2 was demonstrated and it was shown that this interaction depends on the SARAH domain. SARAH domain-deleted RASSF1A had a similar growth-reducing effect as full-length RASSF1A and inhibited anchorage independent growth of the lung cancer cell lines A549 significantly. In cancer cells expressing the SARAH deleted form of RASSF1A, reduced mitotic rates (*P* = 0.001) with abnormal metaphases (*P* < 0.001) were observed and a significantly increased rate of apoptosis was found (*P* = 0.006) compared to full-length RASSF1A. Although the association with microtubules and their stabilization was unaffected, mitotic spindle formation was altered by deletion of the SARAH domain of RASSF1A. In summary, our results suggest that the SARAH domain plays an important role in regulating the function of RASSF1A.

## 1. Introduction

The Ras association domain family 1 gene (*RASSF1)* was identified on chromosome 3p21.3, a region frequently deleted in cancer [1]. There are two major transcripts of *RASSF1*, termed *RASSF1A* and *RASSF1C*, which are transcribed from different CpG island promoters [1]. The promoter of *RASSF1A* is often hypermethylated in cancer, whereas the promoter region of *RASSF1C* is never methylated [2, 3]. Both isoforms encode a Ras association domain in the C-terminus, an ATM-kinase phosphorylation site, a SARAH protein interaction domain, and the N-terminal sequence of *RASSF1A* harbors a diacyl glycerol binding domain [1, 4]. It has been demonstrated that *RASSF1A* encodes a tumor suppressor gene, which reduces tumor growth *in vivo* and *in vitro* [1, 5–8]. Deletion of *Rassf1a* in mice significantly increased spontaneous and induced tumorigenesis [9–11]. It has been reported that RASSF1A binds to microtubules and protects cells from microtubule destabilizing agents [7, 12–15]. This interaction contributes to cell cycle regulation and mitotic progression.

 RASSF1A is regulated by the binding of RAS and the novel Ras effector 1 (NORE1) and mediates proapoptotic signals through binding of the mammalian sterile 20-like kinase 1 and 2 (MST1 and MST2) [16–19]. Moreover, an association of RASSF1A with the BH3-like protein modulator of apoptosis was observed and this interaction regulates conformational change of BAX and apoptosis [20, 21]. RASSF1A promotes MDM2 self-ubiquitination and prevents p53 degradation [22]. Additionally, it was reported that RASSF1A inhibits the anaphase promoting complex (APC) through its binding to CDC20 and induces mitotic arrest by stabilizing mitotic cyclins [23] and it was further shown that the Aurora mitotic kinases are involved [24]. However, we were not able to verify the interaction between RASSF1A and CDC20 [25].

In the C-terminal part of RASSF1A and RASSF1C, a protein-protein interaction domain called SARAH (Sav/RASSF/Hpo) has been determined [26]. The SARAH domain is a key feature of the Hippo signaling pathway components, by which the interaction of Sav, Rassf, and Hpo is accomplished [26]. In the Drosophila Hippo pathway, Salvador (Sav, the human homologue is named WW45) acts as a scaffold protein that interacts with the proapoptotic kinase Hippo (Hpo, human homologue MST) [27–29]. Hpo is able to phosphorylate the kinase Warts (human homologue LATS), which in *Drosophila* leads to cell cycle arrest and apoptosis [28–30].

It was shown that the single *Drosophila* orthologue of the human RASSF proteins restricts Hpo activity by competing with Sav for binding to Hpo [31]. Praskova et al. previously showed that human RASSF1A interacts with MST1 through the C-terminus [16] and more precisely through the SARAH domain [32]. MST1 has two caspase 3 cleavage sites and both MST1 and MST2 play a role in processes of apoptosis both before and after caspase cleavage [33]. The cleaved form of MST1 translocates in the nucleus and phosphorylates histone H2B at Ser14 [34, 35]. H2B phosphorylation correlates with apoptotic chromatin condensation and nuclear fragmentation in mammalians and yeast [35, 36]. Following death receptor activation, MST1 (homologue of Hpo) is known to become activated through caspase-dependent cleavage [19]. The cleaved fragment then localizes from the cytoplasm to the nucleus, where it induces apoptosis [19] by chromatin condensation through activation of the c-Jun N-terminal kinase pathway [37].

Both RASSF1A and WW45 activate MST2 by promoting its autophosphorylation [38]. Moreover, RASSF1A stabilizes MST1/2 activation by preventing the dephosphorylation of these kinases [39]. Activated MST1/2 phosphorylates different targets including LATS kinases, which in turn activate the transcription coactivator YAP1 [40, 41]. Other MST1/2 targets are H2AX [42], FOXO [43], and troponin [44].

To gain new insights into the tumor suppressor function of RASSF1A, we deleted its SARAH domain and analyzed its altered function. Deletion of the SARAH domain resulted in a decreased colony formation of tumor cells. During mitosis, abnormal spindle formation was observed. We demonstrate that the interaction of RASSF1A and RASSF1C with MST1 and MST2 depends on the SARAH domain. Deregulation of the SARAH domain may contribute to altered proapoptotic and mitotic signaling of RASSF1A.

## 2. Materials and Methods

### 2.1. Tissues and Cell Lines

The localization experiments and the protein expression experiments were performed in HEK293 and COS7 cells (ATCC, Manassas, Virginia, USA). For stable transfection, the lung cancer cell line A549 (ATCC) was used. A549 cells harbor epigenetic silenced RASSF1A, but express RASSF1C [1].

### 2.2. Interaction Studies Using the Yeast Two-Hybrid System

The Matchmaker Two-hybrid system (Clontech, Mountain View, USA) was utilized. cDNAs of RASSF1A and RASSF1C were described previously [1]. The genes MST1 and MST2 were cloned after amplification of the fragments from EST-clones IRAKp961C0282Q and IRAKp961I0613Q (RZPD, Berlin, Germany), respectively. RASSF1AΔSARAH and RASSF1A were cloned into the vector pGADT7, RASSF1C and RASSF1CΔSARAH into the vector pAS2-1, and MST1 and MST2 into pGBKT7 [25, 45]. Mutant forms of RASSF1 were generated with the QuickChange XL Site-Directed Mutagenesis Kit (Stratagene, La Jolla, USA) and forward primer (5′-AGGAAAATGACTCTGGGCCCCTTGGGTGACCTCT) and the complementary reverse primer. All constructs were confirmed by sequencing. The yeast strain PJ69-4A was cotransformed with 0.1 *μ*g of each plasmid using the PEG/LiAc method. The interaction analysis was carried out on SD minimal medium plates without adenine and histidine and the transformation efficiency was controlled on SD plates with adenine and histidine. The strength of interaction was investigated by quantification of the expression of the *β*-galactosidase reporter gene with o-nitrophenyl-*β*-D-galactopyranoside (ONPG) as substrate at 420 nm.

### 2.3. Interaction Studies by Coprecipitation

MST1 and MST2 were cloned into the vector pCMV-Tag1 (Stratagene, La Jolla, USA) and/or in the vector pEBG. To investigate the interaction of specific RASSF1 forms, MST1 and MST2, cotransfections (Lipofectamine 2000, Invitrogen, Carlsbad, USA) were performed in HEK293 cells. Plasmids (pEBG and pCMV-Tag 1) were used, that express GST-Flag-RASSF1A, GST-Flag-RASSF1C, GST-Flag-RASSF1AΔSARAH, GST-Flag-RASSF1CΔSARAH, and GST or Flag-MST1, Flag-MST2, and Flag-WW45 [45]. Two days after transfection, total protein was extracted in RIPA buffer. The GST-fused proteins were precipitated with glutathione-sepharose (Amersham Biosciences, Freiburg, Germany). Samples were separated on a 10% PAGE gel and blotted. The interaction was determined with anti-Flag-antibodies (F3165, Sigma, Steinheim, Germany) and anti-GST antibodies (Santa Cruz, Santa Cruz, USA).

### 2.4. MST1 and MST2 Phosphorylation

A549 were treated with 3 *μ*M staurosporine for 3 h or transfected with 10 *μ*g of constructs with Turbofect for 36 h (Fermentas, St. Leon-Rot, Germany). Total protein was isolated using Flag-lysis buffer, samples were denatured with Laemmli-buffer, separated in 10% SDS-PAGE, and blotted onto PVDF membranes. First antibodies are: anti-GAPDH (FL332 Santa Cruz, USA), anti-P-MST1 (Thr183)/MST2 (Thr180) (#3681 Cell signaling, Frankfurt, Germany), anti-Flag (F3165 Sigma, Steinheim, Germany), and secondary antibodies are HRP coupled (sc2004/5 Santa Cruz, USA). ECL (WBKLS0100 Millipore, Schwalbach, Germany) was used for detection with Versadoc (Bio-Rad, München, Germany).

### 2.5. Generation of Stable Transfected Cell Lines

RASSF1A, RASSF1AΔSARAH, and RASSF1C were cloned into the vector pCMV-Tag1 (Stratagene, La Jolla, USA). The lung cancer cell line A549 was transfected using Lipofectamine 2000 (Invitrogen, Carlsbad, USA). Colonies were selected under 1 mg/mL Geneticin (Gibco, Karlsruhe, Germany) in DMEM and clones were picked after 4 weeks. Expression of Flag-RASSF1 was confirmed by RT-PCR using the FLAG-specific primer (5′-TGGATTACAAGGATGACGACG) and RASSF1-specific primer L27111 (5′-TCCTGCAAGGAGGGTGGCTTC). PCR products were analyzed on a 2% Tris-borate EDTA agarose gel.

### 2.6. Proliferation Analyses of Stable Transfected Cell Lines

 Growth curves of stable transfected clones were analyzed by seeding 150,000 cells in triplicates in 6-well plates. Every 24 hours, cells were counted using a Neubauer counting chamber. In order to investigate the proliferation in soft agar, stable transfected cells were seeded in 0.3% agarose. Experiments were performed in duplicates with 5,000 cells per plate under selection with 1 mg/mL Geneticin. Colony size was measured after 4 weeks with a microscope (LEICA DMIRB, Wetzlar, Germany). Therefore, colonies were stained with 400 *μ*L of 5 mg/mL INT and the size of 25 colonies was determined with MetaVue (Molecular Devices GmbH, Ismaning, München).

### 2.7. Localization Studies

RASSF1A and RASSF1C were cloned into the fluorescence vector pEYFP-C2 (Clontech, Mountain View, USA). The deletion of the SARAH domain of RASSF1A and RASSF1C was accomplished by the QuickChange XL Site-Directed Mutagenesis Kit (Stratagene, La Jolla, USA) with the upper SARAH deletion primer 5′-AGGAAAATGACTCTGGGCCCCTTGGGTGACCTCT and the complementary lower primer. After transient transfection into HEK293 cells with Lipofectamine 2000 (Invitrogen, Carlsbad, USA), the localization of YFP-RASSF1A, YFP-RASSF1AΔS, YFP-RASSF1C, and the vector control were investigated with a fluorescence microscope. Cells were co-stained with anti-*α*-tubulin (Molecular Probes, Invitrogen, Carlsbad, USA) antibodies and Alexa Fluor goat anti-mouse (Molecular Probes, Invitrogen, Carlsbad, USA) antibodies to show the colocalization with the microtubules and spindle poles, respectively. Nuclei of the cells were visualized by staining with DAPI (0,1 *μ*g/mL in PBS). Cells in mitoses were scored by microscopy (ZEISS Axioplan 2). Cells with highly condensed chromosomes and spindle structures were classified as mitotic cells. For microtubule stability analysis, the cells were treated one day after transfection for one hour with 20 *μ*M nocodazole, fixed, and stained with DAPI and anti-*α*-tubulin antibodies. YFP constructs are shown in green color.

### 2.8. Apoptosis Analysis by TUNEL Staining

The lung cancer cell line A549 was transiently transfected with different RASSF1A constructs tagged with yellow fluorescence (pEYFP-C2) using Lipofectamine 2000 (Invitrogen, Carlsbad, USA). After two days, the transfected cells were harvested and centrifuged on a slide. After fixation with formaldehyde, a TUNEL staining was performed with the *In Situ* Cell Death Detection Kit, TMR red (Roche, Mannheim, Germany). The nuclei were costained with DAPI (0,1 *μ*g/mL) solution. Slides were quantified with a fluorescence microscope (ZEISS Axioplan 2). Yellow fluorescence expressing cells (500) were counted and the rate of apoptotic cells (red fluorescence) was calculated. All experiments were done in triplicates.

### 2.9. Statistical Analysis

All statistical evaluations were performed using the SPSS 12.0 Software (SPSS Science, Chicago, IL). A Probability of *P* < 0.05 was considered as significant.

## 3. Results

### 3.1. The RASSF1-SARAH Domain Binds MST1 and MST2

**Figure 1:**
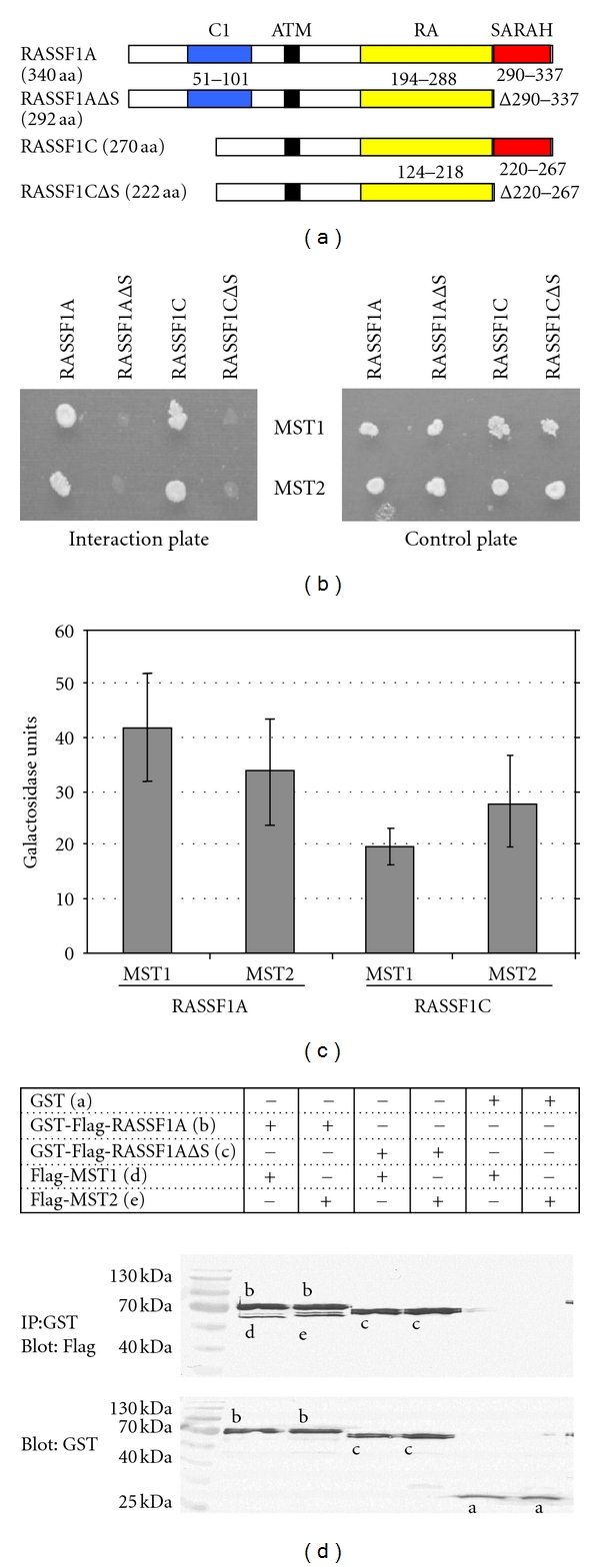
Binding studies of RASSF1, MST1, and MST2. (a) Characteristic domains of RASSF1 isoforms and SARAH deletion (ΔS) mutants are the protein kinase C conserved region (C1; blue), the ATM-kinase phosphorylation site (black), Ras-association (RalGDS/AF-6) domain (RA; yellow), and the Sav-RASSF-Hpo interaction site (SARAH; red). (b) Interaction analysis using the yeast two hybrid system. The indicated constructs were cotransformed into yeast strain PJ 69-4A. Interaction was evaluated on SD plates without alanine and histidine (interaction plate). Transformation was controlled on SD plates with alanine and histidine (control plate). (c) Quantitative interaction analysis using the ONPG assay. In three independent colonies, the activation of the *β*-galactosidase reporter gene was quantified with ONPG as substrate. The standard deviation is indicated. (d) Binding studies in coprecipitation. Constructs that express GST (a), GST-Flag-RASSF1A (b), GST-Flag-RASSF1AΔS (c), Flag-MST1 (d), or Flag-MST2 (e) were transfected into HEK293 cells. After two days, total protein was extracted and GST-tagged proteins were precipitated with glutathione sepharose. Samples were separated on a 10% PAGE gel and blotted. The precipitated and coprecipitated proteins were determined with anti-Flag-antibodies and anti-GST antibodies.

*In silico* analysis of RASSF1A (340 aa) and RASSF1C (270 aa) by PROSITE (www.expasy.org) revealed the presence of a Sav-RASSF-Hpo (SARAH) domain at their C-terminus (290 to 337 and 220 to 267, resp.) (Figure 1(a)). To gain insight into the function of the SARAH domain of RASSF1, the interaction of RASSF1A, RASSF1C, RASSF1AΔSARAH, and RASSF1CΔSARAH with MST1 and MST2 was investigated in the yeast two-hybrid system (Figures 1(b) and 1(c)). Both RASSF1 isoforms (RASSF1A and RASSF1C) interacted with MST1 and MST2, but when the SARAH domain was deleted the proteins were not able to interact anymore. These interactions were quantified in an o-nitrophenyl-*β*-D-galactopyranoside (ONPG) assay (Figure 1(c)). The interaction of RASSF1A and RASSF1C with MST1 and MST2 was verified by coprecipitation experiments (Figure 1(d) and data not shown). The interaction between RASSF1A and MST1 and MST2 was confirmed (Figure 1(d)). There was also an interaction between RASSF1C and MST1 and MST2 (data not shown). Interaction of RASSF1A and RASSF1C with MST1 and MST2 was abolished, when the SARAH domain was deleted (Figure 1(d) and data not shown).

### 3.2. RASSF1A Constructs with Deletion of the SARAH Domain Inhibit Cell Growth

**Figure 2:**
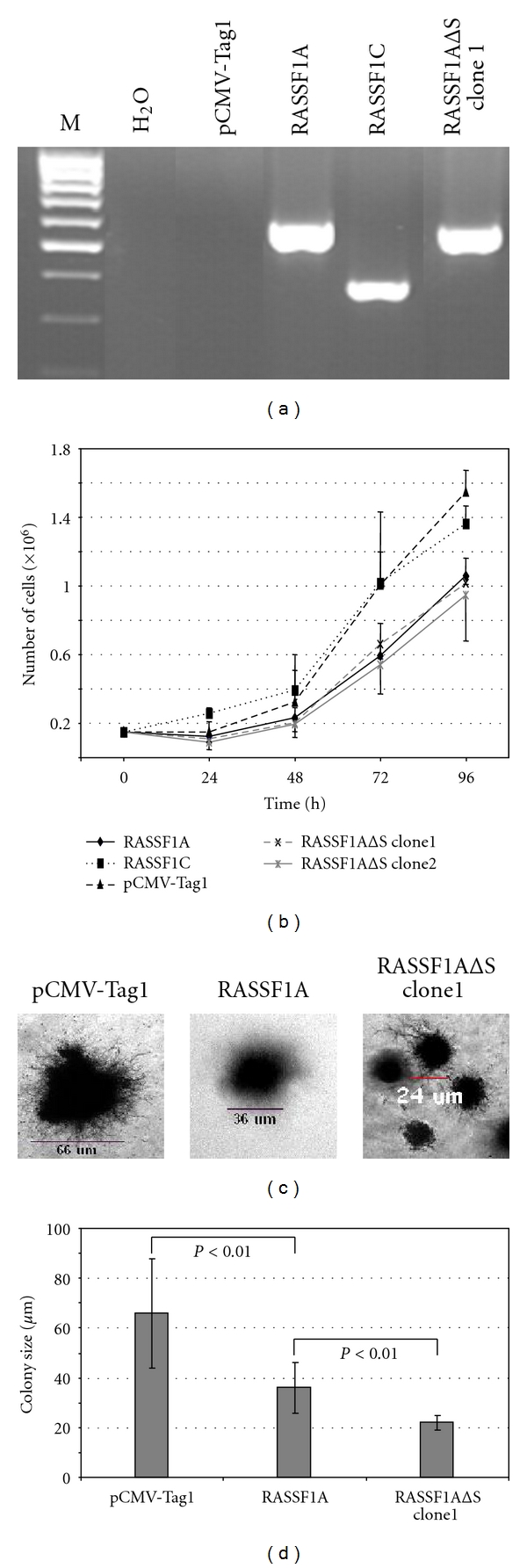
Proliferation analysis of RASSF1AΔSARAH expressing lung cancer cells. (a) A549 lung cancer cells were transfected with pCMV-Tag1, RASSF1A, RASSF1C, and RASSF1AΔSARAH (RASSF1AΔS) and stable clones were analyzed. RASSF1 expression of clones was confirmed by RT-PCR using a Flag-specific forward primer and primer L27111. Products (RASSF1A and RASSF1AΔS, 585 bp; RASSF1C, 374 bp) were analysed with controls (pCMV-Tag1 and H_2_O) and a 100 bp ladder (M) on a 2% Tris-borate EDTA agarose gel. (b) Growth curve of A549 cells stably transfected with the indicated constructs. Clones were analyzed by seeding 1.5 × 10^5^ cells in 6-well plates. Every 24 hours, cells were counted using a Neubauer chamber. Three independent experiments were performed and the mean and standard deviation is plotted. (c) Colony sizes in a soft agar experiment after 4 weeks. Examples of colonies expressing pCMV-Tag1, RASSF1A, and RASSF1AΔS are shown. (d) 25 colonies were measured and the average colony size was calculated. Statistical significance *P*-values are indicated.

Growth effects of the SARAH domain of RASSF1 were investigated in stable transfected lung cancer cells. The lung cancer cell line A549 was transfected with RASSF1A, RASSF1C, and RASSF1AΔS, and the control vector (pCMV-Tag1) and the growth of these cells were evaluated (Figure 2). A549 harbor epigenetically silenced RASSF1A but express RASSF1C. We picked stable transfected colonies and expression of *RASSF1*-specific forms was confirmed by RT-PCR (Figure 2(a)). Subsequently, the proliferation- and anchorage-independent growth of these clones was analyzed. Proliferation of RASSF1A expressing cells was significantly reduced at 96 h compared to RASSF1C expressing cells and control cells (*P* = 0.022 and *P* = 0.007, resp.). Two RASSF1AΔS expressing clones showed a similar growth to RASSF1A expressing cells. RASSF1AΔS clone1 and clone2 had a significant reduction of growth at 96 h compared to RASSF1C (*P* = 0.027 and 0.042, resp.) and controls (*P* = 0.018 and 0.029, resp.). Subsequently, the anchorage independent growth was determined in soft agar experiments (Figures 2(c) and 2(d)). In these experiments, cells expressing RASSF1AΔS (clone1) exhibited a significantly reduced colony growth (average colony size: 22 *μ*m) compared to RASSF1A (average: 36 *μ*m; *P* < 0.01, Welch's test) and control (average: 66 *μ*m; *P* < 0.01, Welch's test).

### 3.3. Expression of RASSF1A with a Deleted SARAH Domain Induces Aberrant Mitosis and Apoptosis

**Figure 3:**
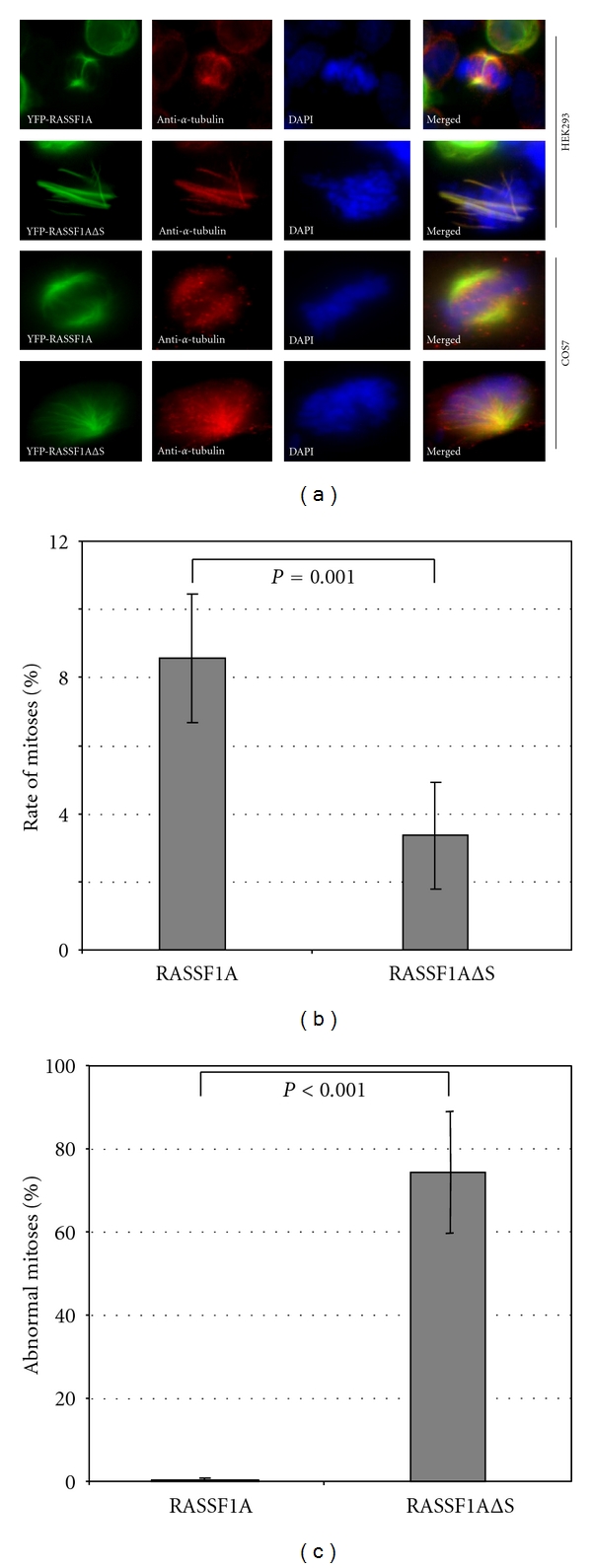
Effects of RASSF1AΔSARAH expression on mitosis. (a) HEK293 and COS7 cells were transfected with YFP-RASSF1A and YFP*-* RASSF1AΔSARAH (RASSF1AΔS) and stained using DAPI and anti-*α*-tubulin antibody. Yellow fluorescent is shown in green. (b) Rate of mitosis in HEK293 after transient expression. Cells with highly condensed chromosomes and spindle structures were classified as mitotic cells (c) Abnormal mitoses (monopolar, multipolar spindles and abnormal spindle fibers) were counted in HEK293 cells and the rate of abnormal mitosis is plotted. All experiments were done in triplicates and 500 cells each were evaluated. The mean and standard deviation were determined. Statistical significant *P*-values are indicated.

We transfected yellow fluorescent protein-tagged RASSF1A and RASSF1AΔSARAH (RASSF1AΔS) into HEK293 and COS7 cells and the localization was determined by fluorescence microscopy (Figure 3(a)). RASSF1A and RASSF1AΔS are both localized at the tubulin-containing cytoskeleton during interphase. In mitotic cells, RASSF1A and RASSF1AΔS were detected at spindles and centrosomes (Figure 3(a)). In the RASSF1AΔS expressing HEK293 cells, multipolar spindles and unequal alignment of the chromosomes between poles were observed (Figure 3(a)). In COS7 cells, overexpression of RASSF1AΔS also induced monopolar spindles (Figure 3(a)). Interestingly, mitotic rate of RASSF1AΔS expressing cells was significantly (*P* = 0.001) reduced to 3.4% compared to 8.6% in RASSF1A transfected HEK293 cells (Figure 3(b)). The mitotic rate in vector transfected cells was 1.2% (data not shown). The majority (74.3%; *P* < 0.001) of mitoses in RASSF1AΔS expressing cells were abnormal (multi- or monopolar) compared to RASSF1A transfected cells, where only 0.6% of abnormal mitosis were counted (Figure 3(c)).

**Figure 4:**
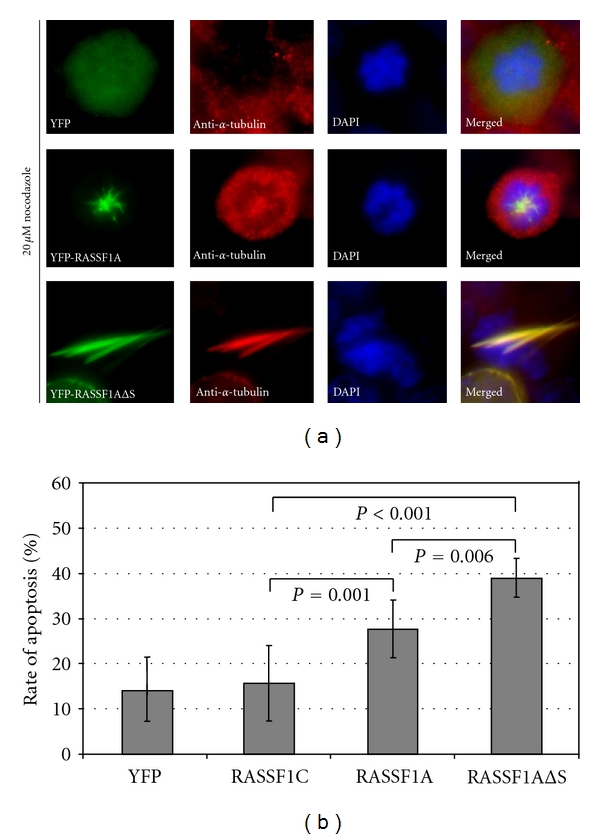
(a) Microtubule stability of RASSF1AΔSARAH expressing mitotic cells. HEK293 cells were transfected with plasmids YFP, YFP-RASSF1A or YFP-RASSF1AΔSARAH (YFP-RASSF1AΔS). One day after transfection, the cells were treated for one hour with 20 *μ*M nocodazole, fixed and stained with DAPI and anti-*α*-tubulin antibodies. Yellow fluorescence is shown in green. (b) Induction of apoptosis after transient expression of RASSF1A and RASSF1AΔSARAH. Lung cancer cells A549 were transfected with YFP, YFP-RASSF1C (RASSF1C), YFP-RASSF1A (RASSF1A), and YFP*-*RASSF1AΔSARAH (RASSF1AΔS). TUNEL staining was utilized to determine the frequency of apoptotic cells in transfected cells. All experiments were done in triplicates and the mean and standard deviation were determined. Statistical significant *P*-values are indicated.

To determine if the aberrant spindle formation in RASSF1AΔS expressing cells is due to an altered microtubule stability of these cells, transiently transfected cells were treated with 20 *μ*M nocodazole for one hour (Figure 4(a)). In YFP control cells, this treatment caused massive depolymerisation of the microtubules in interphase and during mitosis. RASSF1A, RASSF1AΔS, and RASSF1C overexpressing cells were able to stabilize microtubules from depolymerization by nocodazole (Figure 4(a) and data not shown).

To analyze the effect of the deletion of the SARAH domain on the proapoptotic function of RASSF1A, a transient transfection into the lung cancer cell line A549 was performed and the rate of transfected and apoptotic cells was calculated after 1 to 2 days (Figure 4(b)). Apoptotic cells were stained using red fluorescence TUNEL-Kit and YFP was used as a control. The rate of apoptosis was significantly (*P* = 0.001) higher, when the cells expressed RASSF1A (28%) in comparison to RASSF1C (16%) and the YFP control (14%, Figure 4(b)). The deletion of the SARAH domain (RASSF1AΔS) resulted in a significantly increased apoptotic rate of 39% compared to RASSF1C (*P* < 0.001) and RASSF1A (*P* = 0.006, Figure 4(b)). In summary, our results show that expression of RASSF1A with a deletion of the SARAH domain deregulates normal mitotic progression and enhances apoptosis.

**Figure 5:**
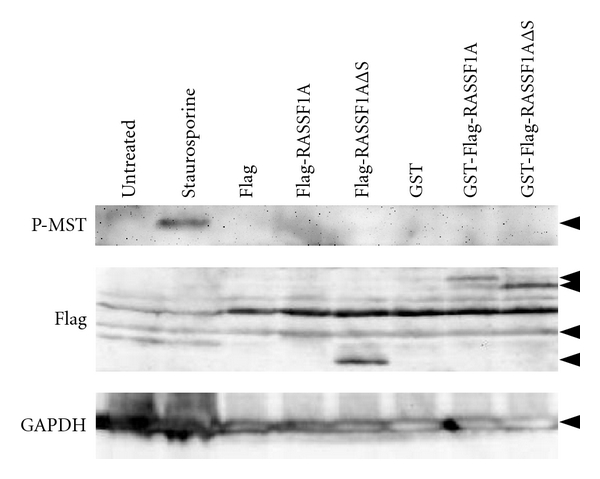
Phosphorylation of MST1/2 is induced by staurosporine. A549 were treated with 3 *μ*M Staurosporine for 3 h. For transfections Turbofect (Fermentas) was used with 10 *μ*g of indicated constructs for 36 h. Total protein was isolated using Flag-lysis buffer, samples were denatured with Laemmli-buffer, separated in 10% SDS-PAGE and blotted onto PVDF membrane. First antibodies are: *α*-GAPDH, *α*-P-MST1 and *α*-Flag and secondary antibody are HRP coupled (sc2004 and sc2005 Santa cruz). ECL (WBKLS0100 Millipore) was used for detection with Versadoc (Biorad). Arrowheads indicate top down: P-MST, GST-Flag-RASSF1A, GST-Flag- RASSF1AΔSARAH (ΔS), Flag-RASSF1A, Flag-RASSF1AΔSARAH (ΔS), and GAPDH.

Subsequently, RASSF1A-induced autophosphorylation of MST1 and MST2 was analyzed in A549 lung cancer cells (Figure 5). For this propose, we have utilized an antibody that detects endogenous MST1 and MST2 only when phosphorylated at Thr183 and Thr180, respectively. Treatment of A549 cells with 3 *μ*M staurosporine induced phosphorylation of MST1 and MST2 (Figure 5), as described previously [34, 46]. However, when A549 cells were transfected with RASSF1A and RASSF1AΔSARAH, phosphorylation of MST1 and MST2 was not detected (Figure 5). Similar results were obtained in HEK293 cells (data not shown). Praskova et al. showed that the MST1 kinase autoactivation through phosphorylation is inhibited by coexpression of RASSF1A and RASSF1C [16].

## 4. Discussion

*RASSF1A* is a tumor suppressor gene, which is involved in several signaling pathway including apoptosis, microtubule stability, proliferation, and mitotic regulation [2, 3]. In our study, we have analyzed the function of the Sav-RASSF1-Hpo (SARAH) domain of RASSF1A. Here, we report that the SARAH domain regulates several pathways, which are frequently altered in tumors. The SARAH domain is involved in apoptosis and growth-suppressing functions of RASSF1A like anchorage-independent proliferation. Moreover, the SARAH domain is important for mitotic progression and spindle formation. It has been previously reported that RASSF1 interacts through its C-terminal domain with MST1 and thereby regulates MST1-mediated apoptosis [16, 18, 19]. We demonstrate that RASSF1A and RASSF1C interact with both MST1 and MST2. This complex may regulate several pivotal signaling pathway including apoptosis and phosphorylation of Warts/LATS serine threonine kinases that regulate mitotic progression.

It has been reported that RASSF1A regulates a proapoptotic pathway through its interaction with the proto-oncogene Ras and the novel Ras effector 1 (Nore1) [18, 32]. RASSF1A and Nore1 interact with the proapoptotic Ste20 protein kinase MST1 via the C-terminus [16, 19] through the SARAH domain [32]. The homologue of RASSF1, NORE1 forms a complex with MST1 that mediates a proapoptotic pathway induced by Ras [18]. Early on, it was reported that MST1 is a serine/threonine protein kinase that could autophosphorylate itself [47] and later Praskova et al. demonstrated that MST1 phosphorylates and activates itself, whereas this autophosphorylation is inhibited when MST1 is bound to RASSF1A and RASSF1C [16]. We show that the interaction of RASSF1A and RASSF1C with MST1 and MST2 depends on the C-terminal SARAH domain. Since the SARAH domain binds the proapoptotic kinases MST1 and MST2, a deregulation of these kinases may contribute to the apoptotic rate in the cells with truncated RASSF1A. However, we could not observe an autophosphorylation of MST1/2 after transfection of RASSF1A and RASSF1AΔSARAH. In contrast, staurosporine induced strong phosphorylation of MST1/2. This indicates that RASSF1A-induced apoptosis observed in A549 cells occurs MST independent, possibly through the N-terminus of RASSF1A, that associates with MDM2 and death-domain-associated protein (DAXX) and contributes to p53 activation in response to DNA damage [22]. Alternatively, RASSF1A was further linked to apoptosis through interacting with the microtubule-associated protein C19ORF5 [48, 49]. Furthermore, Donninger et al. described RASSF1A to interact with the potential tumor suppressor Salvador to promote apoptosis independently of Hippo signaling by modulating p73 [50].

It was demonstrated that RASSF1A colocalizes with the microtubule network during interphase and is found at the spindles and centrosomes during mitosis [14, 23]. RASSF1A binds to tubulin [7], thereby stabilizing microtubules [7, 12–14] and regulating the mitotic progression. RASSF1A overexpression leads to a mitotic arrest at metaphase [14], to a G1 arrest [51], to a G2/M arrest [52], to a G1 and G2/M arrest [7], and a prometaphase arrest [23]. The domain required for both microtubule association and stabilization was mapped to an amino-acid fragment from 120 to 288 [14]. Thus, the microtubule binding site and the SARAH domain are different [12, 14, 53] and this is consistent with our observation. Rong et al. showed that the microtubule binding was lost upon mutation of the phosphorylation site 203 in RASSF1A [54]. However, we and others did not observe an altered microtubule binding using phosphomimicking or nonphosphorylatable mutants of RASSF1A [15, 24].

RASSF1A was also reported to interact with MAP1B (microtubule-associated protein 1B) and C19ORF5 (chromosome 19 open reading frames 5), both microtubule-associated proteins [13, 49, 53]. C19ORF5 is a hyperstabilized microtubule-specific binding protein of which accumulation causes mitochondrial aggregation and cell death [48]. Regarding C19ORF5, it was demonstrated that its knockdown led to mitotic abnormalities [55], that C19ORF5 localizes to centrosomes, and it was stated that C19ORF5 is required for the recruitment of RASSF1A to the spindle poles [53, 55]. Liu et al. reported that RASSF1A caused hyperstabilization of microtubules and the accumulation of C19ORF5 on them [48]. The complex LATS1/MST2/WW45 is found together with RASSF1A at the centrosome, and it was shown that defects in this pathway may lead to abnormal mitosis caused by cytokinesis failure. Thus, RASSF1A may mediate organization of mitotic spindle poles through the recruitment of MST and LATS to the centrosomes.

In summary, our data indicate that RASSF1A is important for several signals, which are frequently altered in tumorigenesis, including apoptosis, mitotic spindle organization, and proliferation. Our data suggest that other domains (e.g., microtubule association domain) than SARAH also significantly contribute to the proapoptotic and antiproliferative function of RASSF1A. Specific interaction of RASSF1A with MST/LATS and other binding partners (e.g., RAS, MDM2, DAXX, C19ORF5, and Salvador) might be important in the regulation of proliferation and apoptosis and in the formation of normal mitotic spindles and processes of dividing chromosomes by RASSF1A.

## Abbreviations

RASSF1A: Ras association domain family 1A
MST: Mammalian STE20 like kinase
LATS: Large tumor suppressor
WW45: 45 kDa WW domain protein
Sav: Salvador
Hpo: Hippo
SARAH domain: Sav-RASSF-Hpo interaction domain
RT-PCR: Reverse transcriptase PCR
YFP: Yellow fluorescence protein
TUNEL: Terminal transferase mediated dUTP nick end labeling.
